# Supplementary figures and images for: Proteomic fingerprinting of Neotropical hard tick species (Acari: Ixodidae) using a self-curated mass spectra reference library
Source: PLoS Negl Trop Dis. 2020 Oct 27;14(10):e0008849. doi: 10.1371/journal.pntd.0008849 (PMC7647123; doi:10.1371/journal.pntd.0008849)

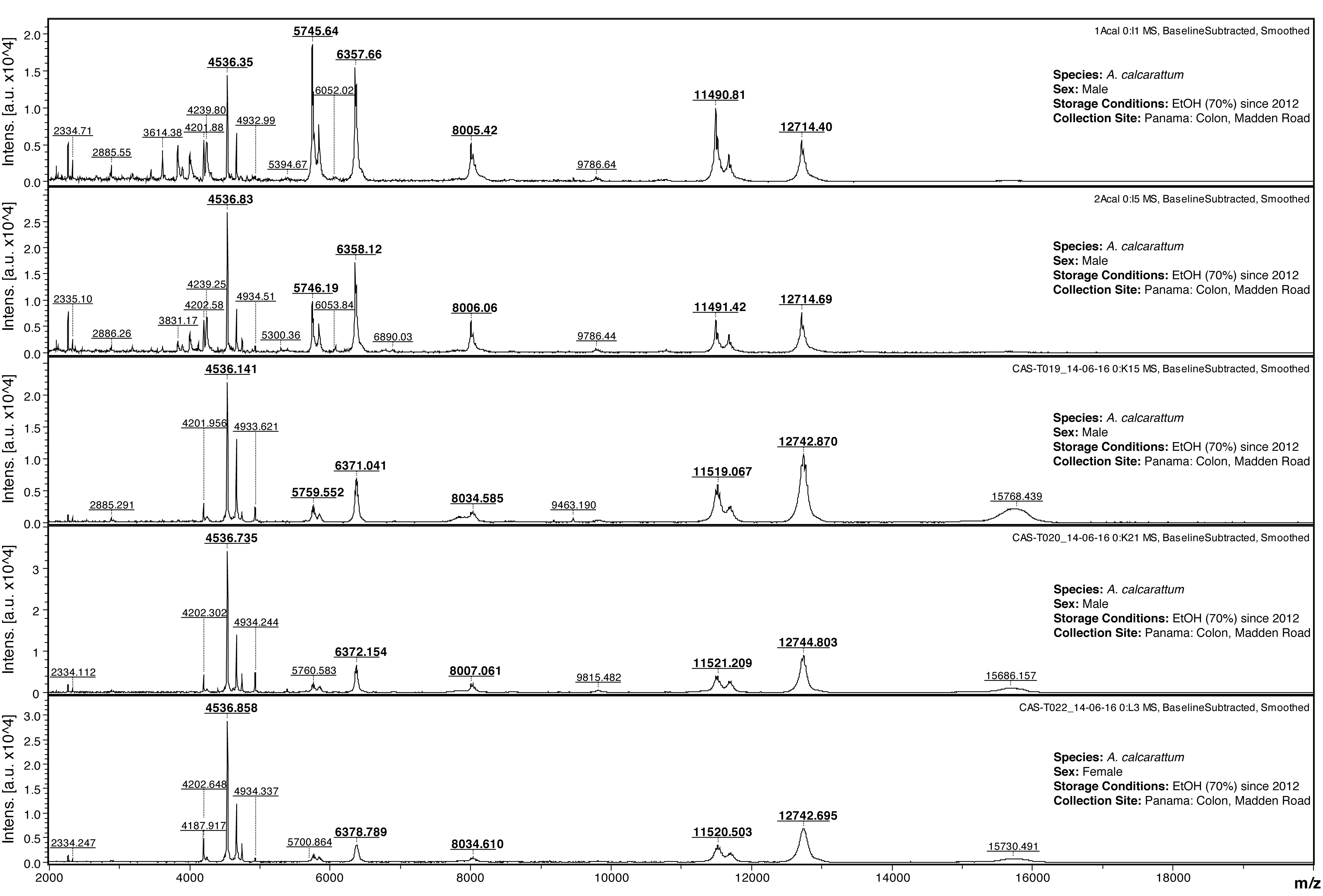

Supplement: S1 Fig — Major ion peaks and their molecular weights are annotated in the range of 2,000 to 20,000 m/z for all specimens. The dataset shows consistently similar protein profiles, regardless of their sex, collection date and/or sampling location. (TIF) [file pntd.0008849.s001.tif]

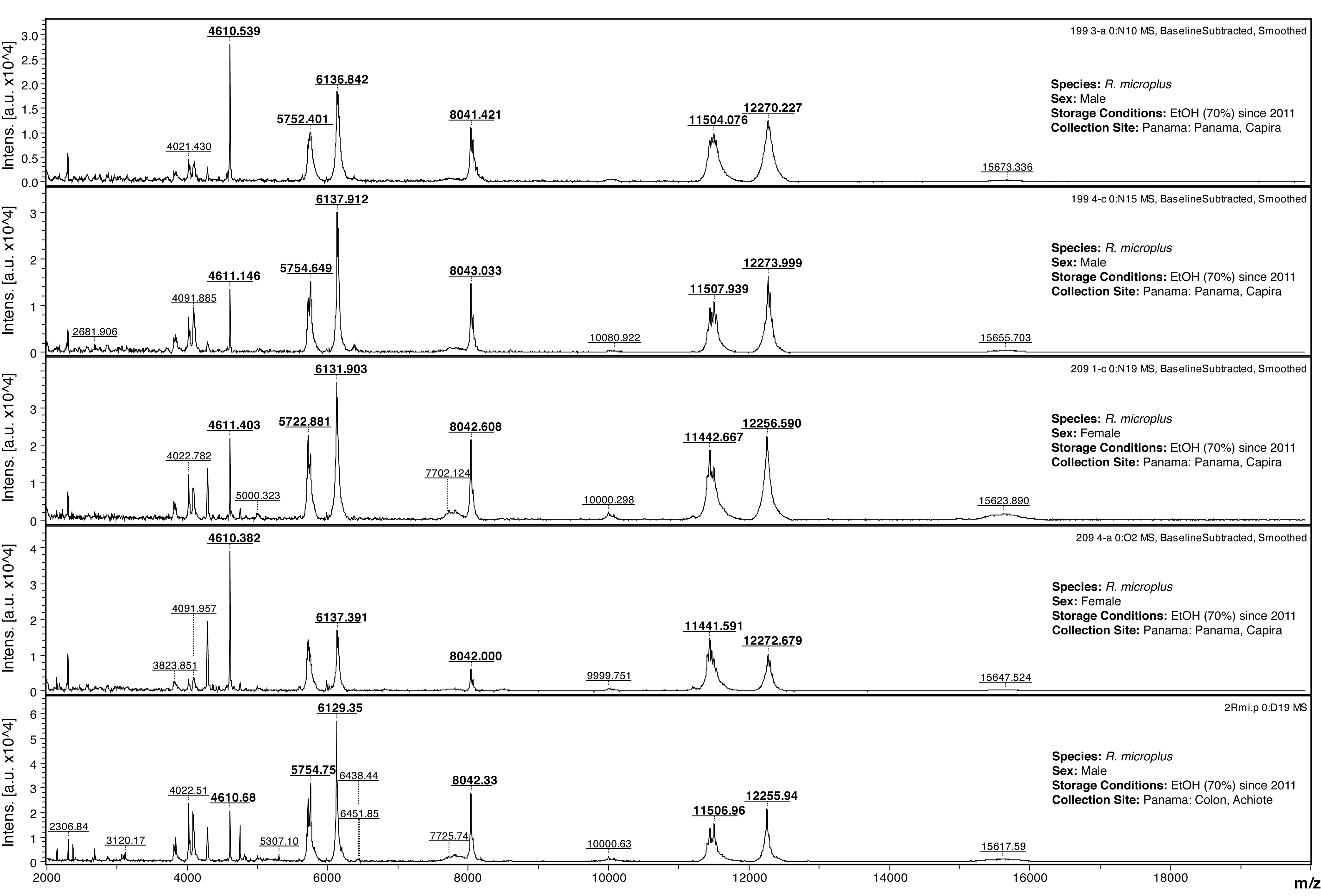

Supplement: S2 Fig — Major ion peaks and their molecular weights are annotated in the range of 2,000 to 20,000 m/z for all specimens. The dataset shows consistently similar protein profiles, regardless of their sex, collection date and/or sampling location. (TIF) [file pntd.0008849.s002.tif]

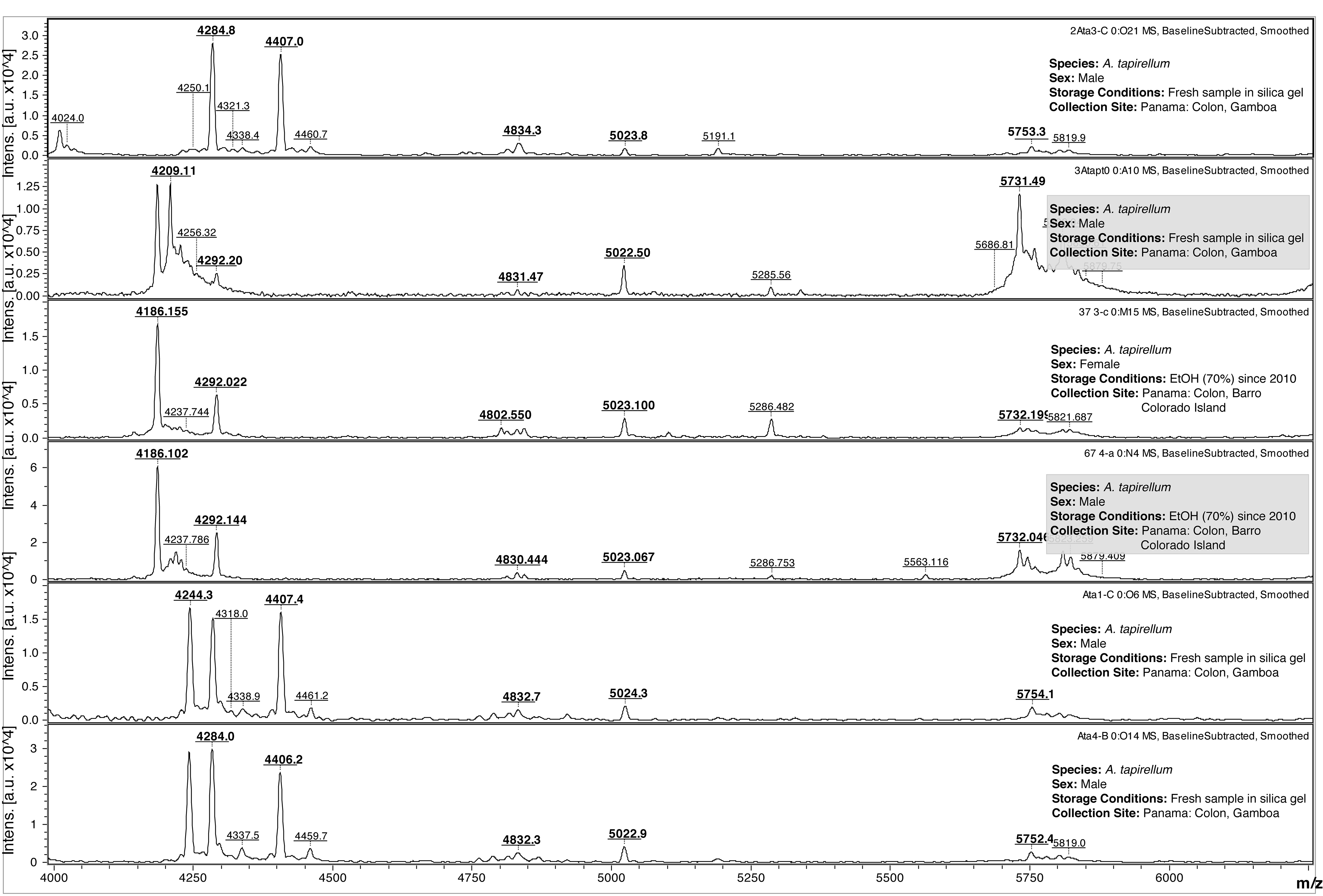

Supplement: S3 Fig — Major ion peaks and their molecular weights are annotated in the range of 2,000 to 20,000 m/z for all specimens. The dataset shows consistently similar protein profiles, regardless of their sex, collection date and/or sampling location. (TIF) [file pntd.0008849.s003.tif]
